# Improvement of 3D Power Line Extraction from Multiple Low-Cost UAV Imagery Using Wavelet Analysis

**DOI:** 10.3390/s19030700

**Published:** 2019-02-08

**Authors:** Anna Fryskowska

**Affiliations:** Department of Remote Sensing, Photogrammetry and Imagery Intelligence, Institute of Geodesy, Faculty of Civil Engineering and Geodesy, Military University of Technology, 00-908 Warsaw, Poland; anna.fryskowska@wat.edu.pl; Tel.: +48-261-83-96-92

**Keywords:** power line inspection, wavelet analysis, low-cost UAV, denoising

## Abstract

Three-dimensional (3D) mapping of power lines is very important for power line inspection. Many remotely-sensed data products like light detection and ranging (LiDAR) have been already studied for power line surveys. More and more data are being obtained via photogrammetric measurements. This increases the need for the implementation of advanced processing techniques. In recent years, there have been several developments in visualisation techniques using UAV (unmanned aerial vehicle) platform photography. The most modern of such imaging systems have the ability to generate dense point clouds. However, image-based point cloud accuracy is very often various (unstable) and dependent on the radiometric quality of images and the efficiency of image processing algorithms. The main factor influencing the point cloud quality is noise. Such problems usually arise with data obtained via low-cost UAV platforms. Therefore, generated point clouds representing power lines are usually incomplete and noisy. To obtain a complete and accurate 3D model of power lines and towers, it is necessary to develop improved data processing algorithms. The experiment tested the algorithms on power lines with different voltages. This paper presents the wavelet-based method of processing data acquired with a low-cost UAV camera. The proposed, original method involves the application of algorithms for coarse filtration and precise filtering. In addition, a new way of calculating the recommended flight height was proposed. At the end, the accuracy assessment of this two-stage filtration process was examined. For this, point quality indices were proposed. The experimental results show that the proposed algorithm improves the quality of low-cost point clouds. The proposed methods improve the accuracy of determining the parameters of the lines by more than twice. About 10% of noise is reduced by using the wavelet-based approach.

## 1. Introduction

Photogrammetric mapping of power lines with the use of photogrammetry is currently a topic of great interest to engineering researchers. It might prove difficult to render the 3D reconstruction of power lines unless appropriate conditions for data acquisition are maintained. Many remotely-sensed data products, such as synthetic aperture radar (SAR), thermal sensor, light detection and ranging (LiDAR), land-based mobile mapping data, and unmanned aerial vehicle (UAV), have been studied for power line surveys [1] and other geosurvey applications [2,3]. Most research papers have focused on the extraction of power lines from aerial laser scanning (ALS) data. Zhu and Hyyppa in [4,5] proposed the use of Automated Power Line Extraction from ALS in forest areas, while Wang et al. in [6] used random forest and neural network algorithms for power line classification in suburban and urban areas. Finally, other elements of energy networks (e.g., pylons) were also modelled from LiDAR point clouds in [7,8]. For all these cases, the other methods RANSAC, Hough transformation and statistical methods were also introduced in [9,10]. Recently, compact and lightweight UAV-LiDAR sensors were introduced into the market, although their performance is limited in terms of scan speed and measurement rate [11]. Ax et al. in [12] presented the use of laser scanner data from a UAV helicopter for vegetation control at high-voltage transmission lines; Teng et al. in [13] proposed their own LiDAR-UAV system. However, most of these systems (LiDAR, SAR, satellite imagery) are quite costly, which is why recent research has focused more on processing UAV imagery data in a way that allows the highest possible accuracy of 3D reconstruction. The linear course of the power lines enforces a specific unmanned data acquisition system. Evidently, corridor mapping will work in this case, especially if multi-rotor platforms are used. The cheaper the platform is, the shorter the flight duration will be, and the worse the optical sensors and navigation systems will be; on the other hand, the costs of purchasing and maintenance of the equipment will be significantly lower. The technology of unmanned systems is constantly getting more advanced and cheaper, which is why more emphasis is put on increasing the accuracy of data processing algorithms than on the types of platforms or the sensors used. There has been very little research on the 3D reconstruction of power lines from point clouds generated from multiple UAV images. The analysis of point clouds from UAV imagery for inspecting power lines was discussed for example in [14,15], where it was applied for detecting cable collision with terrain obstacles. Several researchers have proposed to increase the accuracy of extracting power lines from UAV images by improving the radiometry at the stage of preliminary image processing. In their paper [11], Oh and Lee used horizontal-extended filters to constrain noise and enhance power lines in the image. A similar approach to correcting the digital images and thus improving the resulting quality was confirmed in such publications as [16,17], where the grey ratio operator and mathematical morphology, among others, were used to refine power line feature extraction. 

Improving the image quality plays a significant role in the ability to generate power lines on the image; however, unless certain conditions of image acquisition are met (such as flight altitude, Ground Resolved Distance (GSD), or image overlap), it is not possible to properly generate these data sets. A point cloud is generated from photogrammetric images via image matching in a process called multi-image matching or semi-global matching (SGM). This has been discussed in several publications, for example [17,18,19,20,21], while [22,23] presented the generation of point clouds and the identification of unconventional shapes using video images, and [24,25] proposed the use of oblique images. 

The aim of multi-image matching is to reconstruct the object in 3D by finding homologous points on an image pair with a known inner and absolute orientation [26]. It is difficult to use image-matching methods to find the corresponding points along power lines because of the small diameters of power lines and the complexity of the background [17]. Applying the SGM algorithm results in a point cloud, which is a set of points with XYZ coordinates defined on an orthogonal coordinate system, rendering the object in 3D space. Each point may have additional attributes, such as an intensity value or RGB information assigned from the images [27]. Point cloud quality is directly related to the accuracy of image correlation and depends on factors such as the accuracy of determining tie points on stereo imaging, the geometric complexity of the object, the image block geometry, the radiometric and geometric quality of the images, as well as all stages of image data processing (including interpolation, orientation and others). Point cloud quality was also introduced in [28,29].

Presented in this paper are automatic algorithms to improve point cloud quality with a focus on rendering power lines. The validity of methods was tested in three experimental areas and on the basis of reference data.

### Research Problem

**Figure 1:**
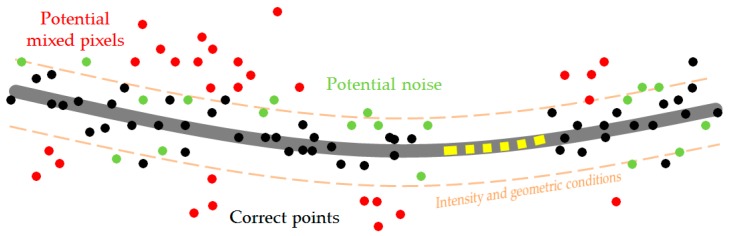
Schematic representation of erroneous points for linear objects.

A typical error in point clouds generated from UAV imagery is noise, most often occurring around the edges, e.g., of roofs, on linear objects (such as power lines) or on the borders between the objects and the background. This phenomenon is often referred to as “mixed pixels”. Mixed pixels in point clouds generated from photographs involve for example the blurring of pixels on the object–background border, leading to disturbances in image correlation, and these are of a slightly different nature than the noise resulting, e.g., from laser scanning [30]. Presented in Figure 1 is a theoretical line and the point cloud representing it. Some of the points can be classified as points representing the object in question—the line (black dots)—while others are either gross errors (mixed pixels—red dots) or errors related to the interpretation of belonging to the object itself (potential noise—green dots). Gross errors are characterised by different intensity values and can also be filtered out by means of statistical analysis and deviation from the average or line approximation. All the other points—those actually representing the object (black) and those included in the measurement noise (green)—may be characterised by similar intensity and are located very close to each other. This causes problems in the interpretation and thus the filtration or classification of a given group of points as “object” or “noise”.

Within the area of potential errors, mixed pixels points, noisy points or real measurement data may also be present. 

The aim of this study is to assess and improve the quality of 3D power line point clouds generated from multiple images acquired using low-cost UAV. The study proposes a method of wavelet-based denoising of UAV-image-based point clouds to eliminate (filter) noisy points. This method involves the application of algorithms for coarse filtration (eliminating gross errors) and precise filtering (eliminating noise) with the use of wavelet transform. Improved point clouds are compared to laser scanning point clouds. A no-reference method for assessing data accuracy is also proposed. 

The paper is structured as follows: in Section 2, the test data are introduced, and in Section 3, the proposed method is explained. Section 4 presents experimental results, followed by the conclusions and discussion in Section 5.

## 2. Materials and Methods

The study uses three data sets obtained via one type of platform (multi-rotor platform). Lines of different diameters were acquired at different heights. In each case, separate images with high overlap (85%) were obtained. Next, the proposed filtering method was implemented in all data sets. 

### Description of Data Sets

In order to carry out a complex analysis of point clouds to reconstruct power lines in low-cost platform images, data on low- and high-voltage power lines were obtained via various photogrammetric systems.

*Set I:* The first data set contains the data obtained with the Riegl platform [31] above the high-voltage lines (pylons *h_T_* ca. 45 m). They were obtained in an open area, locally covered with high vegetation. The diameter of the lines was about 30–40 mm. The data comprised digital photographs and LiDAR data from the Ricopter platform. The photographs were taken with the Sony alpha 6000 camera (with a rigid mount to the VUX-1) with a 24 megapixel 6000 × 4000 matrix and a 16 mm lens. Point clouds from LiDAR were acquired with 380 kHz pulse repetition with the Riegl VUX-1UAV Airborne Laser Scanning system. Power lines were recorded in 3 flight strips at 90 m AGL (Above Ground Level) flight altitude (3 × *h_T_*). The dimensions of the processed section were 670 × 50 m. POSPac MMS 7.1 and RiPROCESS v. 1.6.5 were used for data processing. The point clouds from imaging data were generated in the Pix4D Mapper Pro software with a half-image scale. The data were obtained thanks to the courtesy of the Riegl company. The flight mission took place during the summer. 

*Set II*: The second data set was obtained during a flight mission above low-voltage (0.4 kV) power lines conducted with a Phantom 4 platform in a lowland rural area (Masuria, Poland). The camera mounted on the platform had a focal length of 3.6 mm, 20 Mpix (5400 × 3600) and a Field Of View of 94 deg. The images were obtained in 4 strips at the heights of 23 and 30 m AGL (2,5 *h_T_*). The pylon height was −11 m and the cable diameter was about −6 mm. The point clouds from imaging data were generated in the Pix4D Mapper Pro software with a full-image scale. The data were obtained during summer and winter. Differences in this measurement campaign will be revealed in the experiments.

*Set III*: The third data set comprised low-voltage (0.4 kV) power lines and pylons located in an urban area (Warsaw, Poland). The images were obtained in 3 strips at the height of 28 m AGL. The platform used was DJI Phantom 4 Pro with a 20 Mpix camera and a 3.6 mm focal length. The pylon height was 11 m, and the cable diameter equalled 28 mm in total (5 cable cores). The point clouds from imaging data were generated in the Pix4D Mapper Pro software with a full-image scale.

*Set IV—reference data:* For the purposes of verifying the proposed method, reference measurements were conducted in the form of point clouds from the Terrestrial Laser Scanning system (TLS). Laser scanning was carried out with the Leica P40 impulse scanner [32], which can measure up to 1 million points per second and has an effective measuring range between 0.6 and 200 m (for objects with an albedo of 90%; in practice—about 100 m effective range). The manufacturer certified a 2–4 mm accuracy of determining point coordinates. The scan was made with the target resolution set at 4–5 mm.

An overview of the research data is presented in Table 1.

**Figure 2:**
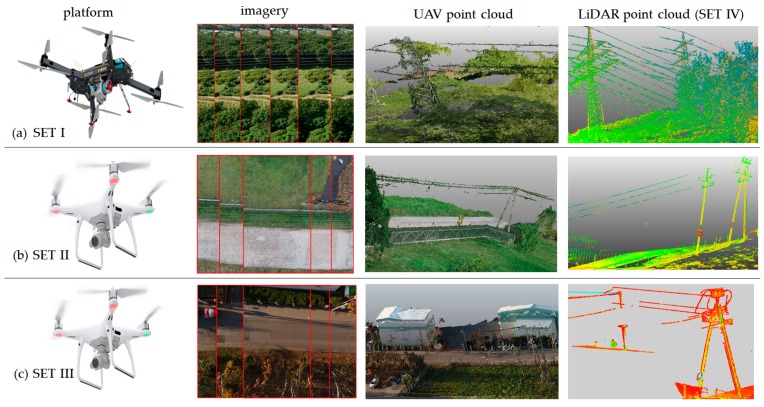
The view for the three data sets: (**a**) forest area and high-voltage power lines: platform, imagery, point clouds (image), point clouds ultra-light light detection and ranging (LiDAR); (**b**) rural area and mean-voltage power lines: platform, imagery, point clouds (image), point clouds (TLS); (**c**) urban area and low-voltage power lines: platform, imagery, point clouds (image), point clouds (TLS).

In each data set, the theoretical diameters of power lines (*ϕ_T_*) did not exceed the range between 6 and 35 mm. The flight height (linked with the GSD value) was always chosen so that the lines were visible on at least 2–3 pixels. TLS data were also obtained from a distance, so that the footprint (laser diameter) was smaller or equal to the cable cross-section width. The data are presented in Figure 2. 

## 3. Methods

**Figure 3:**
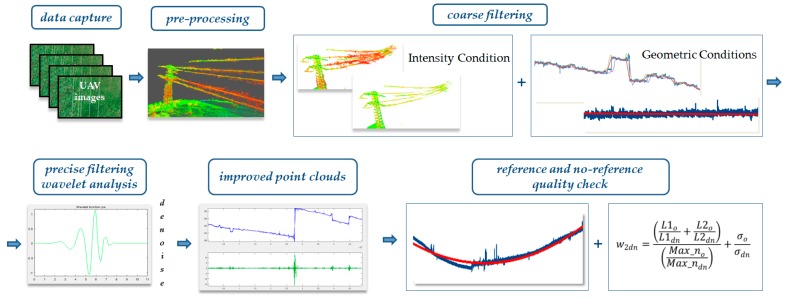
Block diagram of the overall method.

Presented in this chapter is the two-stage method for improving the quality of point clouds by filtering them with the use of wavelet analysis. A block diagram of the overall method workflow is shown in Figure 3.

The proposed approach comprises the stages of data preparation, coarse filtration and precise denoising as well as a quality check based on reference measurements and denoising statistics via wavelet transform.

### 3.1. Data Capture

**Figure 4:**
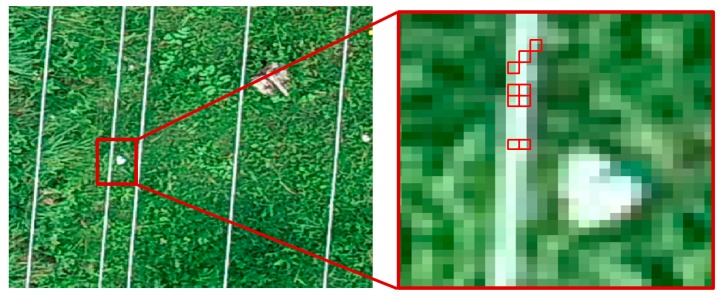
Fragment of the lines and background image for dataset II—GSD 3 mm.

Appropriate acquisition of data makes it possible to detect power lines and is as important as the methods of its subsequent processing. Low-cost UAV systems offer the option of automatic flight mission, both by way of photogrammetric strips and corridor mapping. When acquiring data on power lines via UAV, it is recommended to plan the flight route and strip distribution along the transmission towers. Thus, multi-rotor platforms with flight direction independent from the wind are the preferred UAVs for power line mapping. However, in order to obtain usable imaging data from UAV, it is necessary to adjust the flight parameters and exposure according to the model and type of camera mounted on the platform. The fundamental aspect of acquiring images for power lines inspection is the image resolution from which they are to be extracted. Due to the specific characteristics of the object (power lines can measure less than 1 cm in diameter), the spatial resolution of the images will be important; this is also referred to as the ground pixel value. There are two parameters defining the resolving power of the lens: Ground Resolved Distance (GRD) and Ground Sampling Distance (GSD). The GRD is given by the smallest element distinguishable in the acquired image. Knowing the distance of the sensor from the object, the relation between the image of the smallest object (identified in the photograph, $\Delta x_{i}$) and the real length of the given object is defined by the following, Equation [33]:

(1) $$GRD=H\frac{\Delta x_{i}}{f}$$

where *H* is the distance between the sensor and the object, and *f* is the sensor’s focal length. When mapping linear objects, they must be visible on at least 2 pixels of the acquired image (Figure 4). This is crucial for the algorithms of automatic correlation of images used at the stage of aerial triangulation and generating tie points as well as later when creating a 3D model of the object. 

The GSD—as it is the distance between two consecutive pixel centres related to measurement on the ground—only allows an approximate definition of the interpretation possibilities of the photographs. In turn, the GRD defines the actual resolution of the acquired photograph, taking into account the influences of the optics and the material recording the radiation on image quality [33]. Therefore, the resulting spatial resolution of the images will depend on the flight height and the camera parameters (focal length and pixel pitch). In order to determine the parameters of the flight and images when obtaining data for linear, thin and slender objects (such as power lines), I propose the assumption of the following approximate relation for the optimal flight height, *H*_*FL*[*AGL*]_ (2). This value takes into account the distance of the power line from the ground.

(2) $$H_{FL\left[AGL\right]}=k\cdot \theta \cdot \frac{f}{px}+h_{T}$$

where *h_T_* is the approximate pylon height (distance of power lines from ground), *f* is the focal length, *px* is the pixel pitch, *ϕ_T_* is the theoretical cable diameter and *k* is the thickness coefficient. For low-voltage power lines, *k* = 1, and for high voltage power lines, *k* = 0.5. It should be noted that the planned flight height should be greater than the highest terrain obstacle in the flight area. It is important to take note of the longitudinal and side overlap between the strips, with the recommended value being 80–85%. This will guarantee that the power lines will show up on the stereogram, especially in the case of side overlap. 

Another important element is the georeferencing of UAV imagery. Solving the block adjustment and dense image matching in the local coordinate system leads to smaller adjustment errors, while introducing point transformation into the global coordinate system decreases the accuracy. The acquired images are assigned initial information about their position and attitude from the global navigation satellite systems (GNSS) and inertial measurement unit (IMU). However, in this case, we are only looking at low-cost solutions based on the airborne measurement unit. This paper focuses solely on the quality and consistency of point clouds without taking into account the accuracy of data transformation for the global coordinate system.

### 3.2. Imagery Pre-Processing

**Figure 5:**
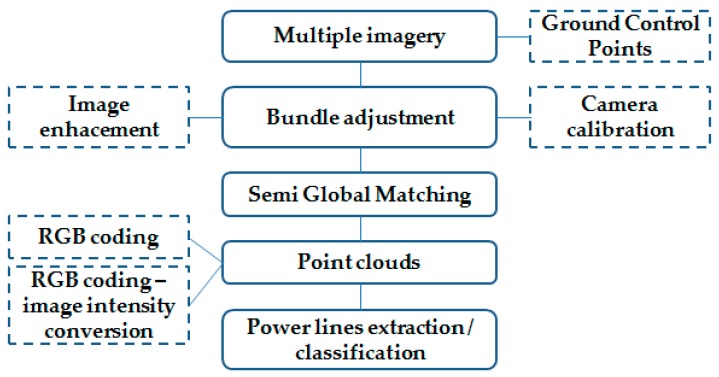
Pattern for generating power lines point clouds from unmanned aerial vehicle (UAV) imaging data (dotted lines indicate optional stages).

All data (photographs) were processed with Pix4Dmapper Pro software, and the point clouds were generated with the use of the Semi-Global Matching algorithm. The process of preparing and generating point clouds is shown in Figure 5. 

Optional stages of data pre-processing include the recommended radiometric enhancement of images before the adjustment stage [17,34] or the calibration of non-metric cameras mounted on the platform [35,36,37]. The final stage of extracting the lines from the point clouds can be carried out via different algorithms, automatically or manually [5,38]. Colour coding the point cloud from the acquired imagery is very helpful when classifying point clouds. The platform-mounted camera is composed of the navigation system, which means that the photographs have defined elements of exterior orientation, i.e., the positions of the projection centre and inclination angles. The mathematical relation between the ground coordinates of points (X,Y,Z) and the position of its image on the photograph (image coordinates x,y) allows us to determine the position of every point with (X,Y,Z) spatial coordinates on the photograph (x,y), and from this place, we can obtain colour information in the form of RGB components that have the integer range [0; 255]. 

**Figure 6:**
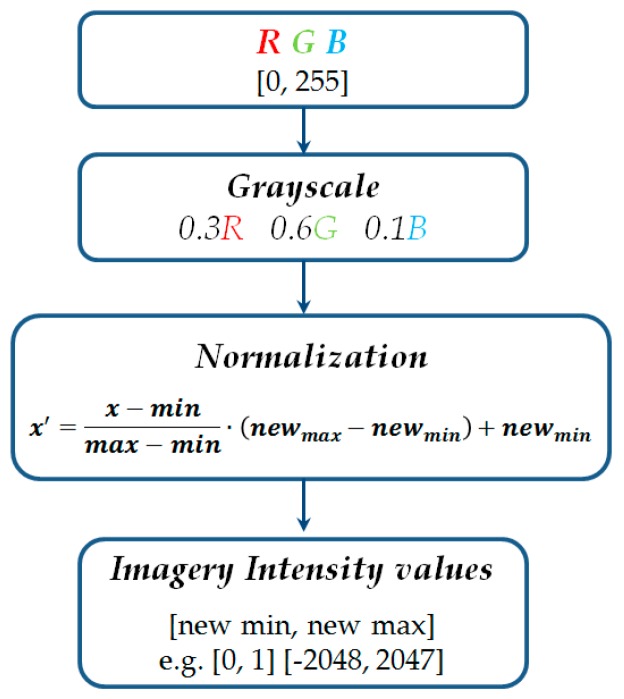
RGB to imagery intensity scale conversion scheme.

In the case of point clouds generated from UAV imagery, instead of an RGB value, a point may be assigned a new value—the image intensity (*I_IMG_*). The conversion scheme is presented in Figure 6. 

RGB values for every point can be converted to grayscale by weighing the Digital Number (DN) value for every channel and then normalised to the intensity scale expressed for example in the ranges: [0; 1], [−2048; 2047] or others, depending on the file format. Such normalised data will provide a basis for further operations with the algorithm. Intensity scale was used in this research. 

### 3.3. Classifying and Filtering Power Lines 

Power lines are situated parallel to each other, anchored at both ends to the pylons. This constitutes a defined and geometrically repeatable arrangement of objects, which may be helpful when classifying points belonging to the ground, vegetation, buildings or other classes. The classification process itself is quite complex and can be conducted in different ways. Among the available methods, examples of power line classification were introduced in [12,38]. Single-line separation algorithms are very helpful, for example, Spatial Clustering introduced in [9] and splitting pans in [5]. Most sources discuss the classification and filtering of data obtained via LiDAR systems, which makes the task considerably easier. Point clouds generated from UAV imagery are significantly noisier, with a strong presence of mixed pixels. Discussed below is the proposed two-stage approach to filter point clouds from UAV imagery. 

#### 3.3.1. Two-Stage Coarse Filtration

The first stage of data processing is coarse filtration, which results in the elimination of gross errors and any points significantly departing from the studied object (cable) which prohibit a correct detection of its shape. The proposed initial filtering has to do not only with the shape of the line, but also with the attributes of the points, i.e., the RGB value or imagery intensity assigned to each point.

Coarse filtration mostly concerns gross errors and mixed pixels resulting from dense image matching. It may be based on the statistical analysis of imagery intensity or the values from the R, G and B channels. Therefore, the filtering will constitute the result of, e.g., segmentation or dividing the points according to a certain pattern. The basic assumptions that have to be met for the segmentation/thresholding of point clouds are, firstly, the diversity of the intensity values and secondly, the existence of a relation between a particular object/class and intensity. One of the filtering methods is thresholding. The basic assumption for thresholding is the extraction of the object from its background by assigning appropriate intensity values. When segmenting point clouds with the use of thresholding, the points are divided into those for which a set value of imagery intensity (*I_IMG_*) in relation to the neighbouring points falls below the threshold and those in which the value is equal to or higher than the threshold value, *t*:

mixed pixels *for I_IMG_ < t*

accepted points *for I_IMG_ ≥ t*

The above relation assumes that points with a high (above-threshold) intensity are accepted as points containing the lines and noise, while pixels with low intensity are gross errors. With global thresholding methods, the threshold value remains fixed for the entire data set and refers to every point. The most difficult to define is the threshold value, *t*, i.e., the condition for which points are considered noise or mixed pixels and which are not. In the case of mapping power lines, it is especially difficult, because it depends directly on presenting the lines on original (source) images and the relation between the image of the lines and the background or surroundings. The lines on the point clouds generated from the photographs will “take on” the noise resulting from the process of correlating the image. 

There are several methods of automatic thresholding, including statistical methods, global and local thresholding with iterative methods, linear combination, average and standard deviation, and Otsu algorithm described in [39,40,41].

The most frequent problem with the majority of thresholding methods is that the main parameter taken into consideration is the intensity (both with imagery and LiDAR data), and not, e.g., topological and geometric relations between neighbouring pixels or points. That is why there is no certainty that the objects identified as background objects in the thresholding process are of a continuous nature. This can result in including points from outside the class or excluding certain correct points, especially around the object–background border. The stronger the data noise is, the more these effects intensify, and the more probable that the intensity or colour of the points does not reflect the average intensity for the given class. This is clearly visible in Figure 1 (mixing of green and black points). Thus, the greatest challenge for effective thresholding lies in determining the threshold value *t*. Consequently, setting the threshold leads to losing “good” data from the object and intensifying the “bad” data from the background. The phenomena described above are common and troublesome when filtering UAV data, especially in the case of power lines. 

The proposed two-stage method for the coarse filtration of power lines from point clouds is based on Sauvola and Pietikäinen’s thresholding algorithm [42] in terms of intensity and geometric conditions.

##### Intensity Condition

In the part concerning intensity filtering, the threshold value is determined based on the average and standard deviation of point values in the range directly surrounding the analysed point:

(3) $$t=\mu \left[1+k\cdot \left(\frac{\sigma }{\sigma_{max}}-1\right)\right]$$

where *μ* stands for the average, *σ* is the standard deviation of the point intensity in the surroundings, *k* is a parameter depending on the data (*k* = 0.2 for very noisy data, *k* = 0.15 for medium noise, and *k* = 0.07 for low noise), and *σ_max_* is the dynamic range of standard deviations for the data. The criterion for the level of noise of the data is the relation between the values of the standard deviation and the average. 

As a result of segmentation based on intensity values, there still remain some points whose intensity fits within the threshold value but that are located outside of the desired object, i.e., gross errors of this stage of filtering. That is why a second stage is necessary: segmentation with geometric conditions.

##### Geometric Conditions

Due to the fact that power lines must be horizontal straight lines (projected to the xy-plane), and vertically (projected to the z-axis), i.e., catenaries, it is necessary to conduct filtering that takes these trends into account. The first stage is filtering by horizontal coordinates to analyse the approximation of the linear trend (Algorithm 1), while the second occurs on the Z coordinate, with the analysis of side trends using the moving average (Algorithm 2).


**Algorithm 1. Filtering points horizontally**
  *Initial: points from data set after coarse filtration*  **Determining an approximated line based on power line’s points**1:   Look for a linear model for a straight line: *Y_t = α + βt, (t,y_t)* for *t* = 1, …, *n*
2:   Solve the optimisation problem: 
     $\underset{\alpha ,\beta }{min}\left(\overset{n}{\underset{t=1}{\sum }}e_{t}^{2}\right)=\underset{\alpha ,\beta }{min}\left(\overset{n}{\underset{t=1}{\sum }}{\left(y_{t}-\alpha -\beta t\right)}^{2}\right)$

     where $e_{t}=y_{t}-{\overset{˘}{y}}_{t}$ ($y_{t}-$ realisation of variable *Y_t_*), ${\overset{˘}{y}}_{t}=\alpha +\beta t$ (theoretical value of variable)3:   Determine the coefficients defining the straight line:
   $\begin{array}{c} \overset{˘}{\alpha }=\bar{y}-\overset{˘}{\beta }\bar{t}\\ \overset{˘}{\beta }=\frac{\left(\sum_{t=1}^{n}\left(t-\bar{t}\right)\left(y-\bar{y}\right)\right)}{\sum_{t=1}^{n}{\left(t-\bar{t}\right)}^{2}} \end{array}$                  
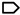
       *straight line is constructed*4:   Calculate the distance of each point *P*(P*_y_*, *P_x_*) from the line d’(P,L)5:   Reject the points whose distance exceeds the set d value constituting 65% of the theoretical radius of the cable
   *d* ≤ 0.65 ⋅ *ϕ_T_*                      
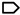
     *output: correct points*

Consequently, the algorithm excludes the points whose distance from the line is bigger than the predetermined value of 65% of the theoretical radius of the cable ϕ_T_. The analysis of side trends for the vertical coordinate Z will work in a similar way. Because of the shape of power lines, the analysis of noise “around” the line, considering it vertically, is not a straight line and does not always have to fulfil the condition of a “regular curve”. Furthermore, in dense image matching, the Z coordinate is the most prone to errors at the stage of data acquisition, which later influences the 3D modelling of the object. To analyse the noise distribution around the Z coordinate, Bollinger Bands were used based on the moving average (MA, determined by means of the simple moving average—SMA) and the analysis of side trends. To this end, it was necessary to define the moving average intervals. The number of intervals depends on the course of the power line and may be determined by the quality of the data. For example, we can determine any constant segments of the power line in a point cloud or places where the line’s course or shape changes as intervals. The value can be set at decimetre-long segments of the line. The Bollinger Bands define the natural extremes in the developing trend. The boundaries of the envelopes located below and above the curve in the constant moving average are expressed as a percentage of the distance, the limits being constructed from the Bollinger Band at a distance equal to a certain number of standard deviations. As the standard deviation depends on the variability of data, the bands adopted for the analyses of power lines should differ from the average by a value of 1σ (65%). When constructing the limits, we should use the one sigma rule, according to which the data will be found between the two bands for 65% of points from the interval. Points located outside of the assumed interval should be rejected. 


**Algorithm 2. Filtering points vertically**
  *Initial: points from data set after coarse filtration*  **Determining Bollinger Bands for power line approximation**1:    Divide the points of the line with the length *dL* into a set number of intervals *n* = 0.1 *d_L_*
2:   Determine the moving average:
      ${\bar{y}}_{t}=\frac{y_{t}+y_{t-1}+\ldots +y_{t-n-1}}{n}$

   for *y*—the variable Z, and *t*—the current points interval, *n*—the number of intervals in the average
   Determine the side trends—upper (UT) and lower (LT):3:   *UT = MA + σ*
   *LT = MA − σ*4:   Reject the points outside side trends.       
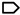
       *output: correct points*


**Figure 7:**
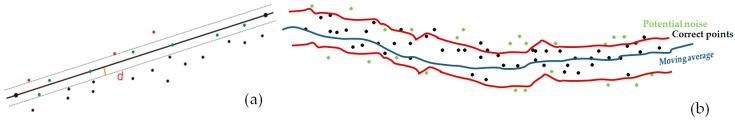
Schematic results of filtering algorithms: (**a**) Algorithm 1, (**b**) Algorithm 2.

The schematic results of the algorithms are presented in Figure 7.

Figure 7a shows how, according to Algorithm 1, points belonging to the horizontal straight line are chosen. A point qualifies (green colour) if its distance from the straight line is shorter than the assumed threshold value. The results of Algorithm 2 are presented in Figure 7b, where the blue colour represents the moving average and red shows the upper and lower bands around this average. 

As a result of the overall coarse filtration process, about 90% of “obvious” noise is removed, leaving the data that, with the use of geometric approximations or simple static analyses of intensity variation, are “unnoticeable” by the proposed algorithms. Therefore, wavelet analysis tools were used for further analysis. 

### 3.4. Denoising Using Wavelet Analysis

**Figure 8:**
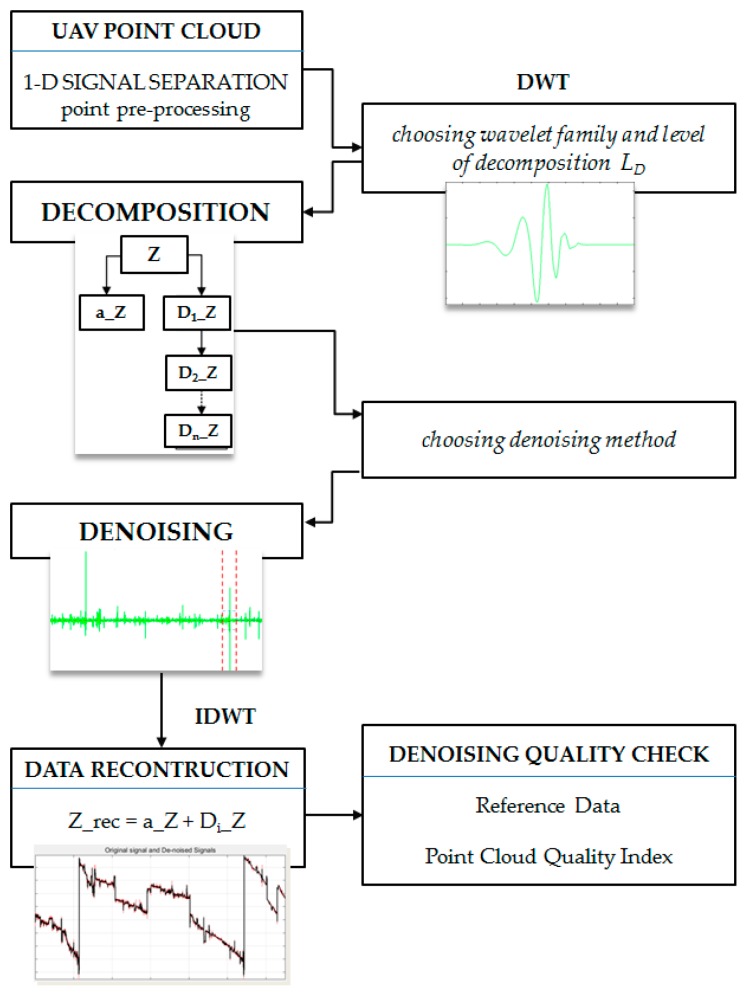
Data denoising workflow, Z—vertical coordinate of UAV imagery point clouds, *D_i_*—details; *a_Z*—approximation of the Z coordinate.

Presented in this section is a novel process for the precise denoising of data representing power lines with the use of wavelet analysis. This requires proper pre-processing of data, defining the wavelet family and choosing a thresholding method. In addition, discussed below is the no-reference method for assessing the accuracy of data filtering. Figure 8 demonstrates the stages of preparation for the filtration process and the process itself. 

In the presented figure, the meanings of the symbols are as follows: Z—vertical coordinate of UAV imagery point clouds, *D_i_*—details, *a_Z*—approximation of the Z coordinate.

#### 3.4.1. Data Preparation

When considering power lines, we focused mainly on the following parameters of the object: span, diameter and sag. This last parameter is the most variable in the long term and depends on factors such as the ambient temperature or wear and tear. Thus, one of the most important coordinates to consider is the Z coordinate (understood as a value on the vertical axis in a rectangular coordinate system). In further steps of the proposed methodology, wavelet analysis will use one-dimensional signals representing the vertical coordinate, arranged in relation to the horizontal Y coordinate. 

#### 3.4.2. Wavelet Analysis

Wavelet analysis is used in many geometric problem solutions and point cloud processing, for example, in [29,43]. Wavelet transform represents a one-dimensional signal *f (t)* as a linear superposition of atomic functions called wavelets. In the case of discrete wavelet analysis, the signal is scaled and shifted discreetly. Then, we express

(4) $$\psi_{j,k}\left(t\right)=\frac{1}{\sqrt{s_{0}^{j}}}\psi \left(\frac{t-k\tau_{0}s_{0}^{j}}{s_{0}^{j}}\right)$$

where *j* and *k* are integers and s_0_ > 1 is the spreading step, on which the shift *τ_0_* (where τ_0_ = 1) depends. Usually, coefficient *s*_0_ = 2. The sampling frequency corresponds to the dyadic sampling.

**Figure 9:**
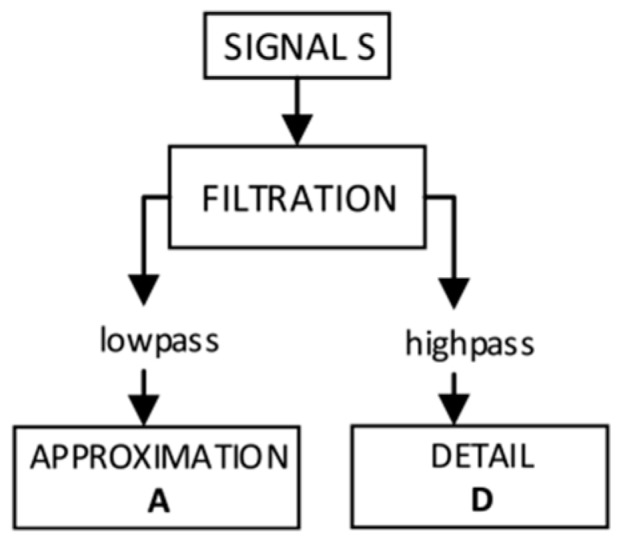
Diagram of wavelet decomposition [45].

In Discrete Wavelet Transform (DWT), the wavelet can be represented as a high pass filter. This issue is used when the wave propagates in a multi-resolution structure. These developments can be determined using a discrete algorithm of a multistage filters. The algorithm based on this dependency is called the Mallat algorithm [44]. The wavelet analysis represents a signal using hierarchical resolution to receive information about the signal that can be presented in different levels of detail. It extracts a general approximation but also enables the selection of some details, disturbances or noise. The signal is decomposed into an approximation (correspond to low-frequency part) and details (represents the high-frequency part). A simple diagram of wavelet decomposition is shown in Figure 9.

Approximation (A) determines the general characteristic of signal tendency, while the details (D) correspond to the high-frequency part and detailed nature of the signal, enabling the separation of noise from the signal. All signals representing individual coordinates for power lines’ data were analysed using wavelet analysis. Each signal (point series) was decomposed to a particular number of levels in different intervals of details and to a component called approximation. Then, by sum of details and approximation, the signal was reconstructed.

1. Choice of mother wavelet and decomposition level

The first task in any wavelet data analysis is to choose a decomposing mother wavelet and the level of this decomposition. There are several ways to set these parameters. Some of them are based on the entropy value of energy distribution and partly on correct energy distribution in frequency bands. In choosing the wavelet function, there are several factors that should be considered: orthogonality, complexity, width and shape [46,47]. A very important parameter is orthogonality, because it implies that the energy content of a signal is preserved through the wavelet transform and it allows for multiresolution analysis (MRA). Furthermore, mother wavelet choice also depends on the waveform shape of the signal (which is especially important for power lines). The chosen mother wavelet should be close to the analysed signal. Hence, it gives a perfect reconstruction with few decomposition levels. 

Decomposition, depending on data, can be single- or multi-level. It is assumed that the level of decomposition is dependent on the length of the signal (series). There are only a few methods which allow the level of decomposition to be defined for signals, and often, it is a theoretical or empirical estimation. The main way to define it is by means of entropy [43].

2. Denoising the wavelet domain—chosen thresholds

After choosing the proper level of decomposition, the type of data denoising has to be proposed. Having conducted several analyses of wavelet characteristics, it was concluded that an orthogonal family of wavelets, such as a symlet or Daubechies wavelet, is a good choice for denoising signals. Furthermore, an orthogonal transform preserves the L2-norm. The basic principle of most wavelet denoising techniques is to modify the wavelet coefficients obtained by the transform before they are reverse transformed [48]. The process of thresholding wavelet coefficients can be divided into two steps: policy choice and threshold function *t*. The choice of the threshold parameter in wavelet function estimation is briefly explained in [26]. There are two basic types of thresholding: hard and soft. A method called soft thresholding, where wavelet coefficients are thresholded and the remaining coefficients reduced by the value of the threshold prior to reconstruction, was proposed in [49]. This was performed using a relatively straightforward threshold, the Universal Threshold, where *L* is the length of the data. Donoho and Johnstone took idea further with new thresholding rules: MiniMaxi, SUREShrink and Hybrid SURE [48,50]. Therefore, the non-linear thresholding function in wavelet domain will tend to keep a few larger coefficients representing the function, while the noise coefficients will tend to reduce to zero [51]. However, it can also be noticed that the detail coefficients *d1* consist mainly of noise, and the standard deviation of the *d1* coefficients is taken to be that of the noise. If orthogonal DWT is used, the risk function *R* can be expressed as

(5) $$R\left(\overset{ˇ}{f},f\right)=\frac{1}{N}\overset{N-1}{\underset{i=0}{\sum }}{\left(\overset{ˇ}{f_{i}}-f_{i}\right)}^{2}=\frac{1}{N}\overset{}{\underset{j,k}{\sum }}{\left(\overset{ˇ}{v_{j,k}}-v_{ij,k}\right)}^{2}$$

where *f_i_* is samples of a deterministic function *f*, $\overset{ˇ}{v_{j,k}}$ are estimated coefficients obtained on the basis of the selected threshold *t* for wavelet coefficients at scale *j:* $t={\left[t_{1},\;t_{2},\;\ldots ,\;t_{J}\right]}^{T}$. If the soft thresholding function is used, nonlinearity is applied to the empirical wavelet coefficients at each scale *j*, *j*−1, …, *j*:

(6) $$n_{s}\left(x,t_{j}\right)=sgn\left(x\right){\left(\left|x\right|-t_{j}\right)}_{+}$$

In research, also subjected to analysis were fixed thresholding, the SURE algorithm and the minimax algorithm. Usually, the thresholds are the same at all scales, but in some algorithms, such as the scale-dependent scheme—SUREShrink, the thresholds at different scales are generally different [51]. The SURE is used only if the level has a significant signal present. Otherwise, universal thresholding is used, which is the simplest threshold (named also fixed threshold). Donoho and Johnstone [49] developed thresholding based on the function $\sigma \sqrt{2\log n}$, where *n* is the sample size and $\sigma^{2}$ is the noise variance. This threshold was one of the first proposed and provides easy, fast and automatic thresholding. The rationale is to remove all wavelet coefficients that are smaller than the expected maximum of an assumed normal noise sequence of a given size [52]. Threshold selection alternatives based on minimising certain optimisation criteria include the minimax [53], and the SURE [49] methods. SURE algorithms are divided into rigorous and heuristic.

### 3.5. Quality Check—Point Cloud Quality Index

After the data has been denoised, the accuracy of the denoising must be checked. If there are no reference data available, the proposed no-reference method of results analysis should be applied. In the case of the no-reference method, the quality coefficients for denoising point clouds proposed by the author in [29] should be used. The quality of point clouds can be determined based on the statistical parameters of signal decomposition and decomposition in the context of data noise. After denoising the data, the statistical parameters of the process are determined. These include L1 norms and L2 norms and are known as least absolute deviation (LAD) errors. It is basically minimising the sum of the absolute differences (S) between the target value (*y_i_*) and the estimated values (*f*(*x_i_*)):

(7) $$S=\overset{n}{\underset{i=1}{\sum }}\left|y_{i}-f\left(x_{i}\right)\right|$$

The L2-norm is also known as least squares. It is basically minimising the sum of the square of the differences (*S*) between the target value (*y_i_*) and the estimated values (*f(x_i_*)):

(8) $$S=\overset{n}{\underset{i=1}{\sum }}(y_{i}-f{\left(x_{i}\right)}^{2}$$

Except for the L1 norm and L2 norm, Max norm and standard deviation values were used. When considering denoised data quality, we should relate to the original values of the signal and its statistics. When analysing results, two wavelet denoising quality indexes are proposed:

(9) $$w_{1dn}=\frac{\left(\frac{L1_{o}}{Max\_n{\;}_{o}}\right)}{\left(\frac{L1_{dn}}{Max\_n{\;}_{dn}}\right)}$$

(10) $$w_{2dn}=\frac{\left(\frac{L1_{o}}{L1_{dn}}+\frac{L2_{o}}{L2_{dn}}\right)}{\left(\frac{Max\_n_{o}}{Max\_n_{dn}}\right)}+\frac{\sigma_{o}}{\sigma_{dn}}$$

Equation (9) presents the ratio of the normalised L1 norm (L1_o_) parameter of the original signal to the normalised L1 norm (L1_dn_) parameter of the signal denoised with a particular threshold method. Equation (10) shows the relation of statistical parameters of coefficients residuals, taking into consideration also the standard deviation of both the original and denoised data *(σ_o_* and *σ_dn_*). The *W_2dn_* index also presents the relation between noisy and smoothened data. Both indexes indicate the relative level of noise in the data.

#### Validation—Comparative Study

Apart from the statistical analysis of data filtering, another important aspect is the parameters of power lines subjected to geodetic measurements. To this end, reference data from laser scanning were used. Quality parameters of point clouds representing power lines adopted for quality check were span, diameter, sag and point cloud resolution (average distance between neighbouring points). 

**Figure 10:**
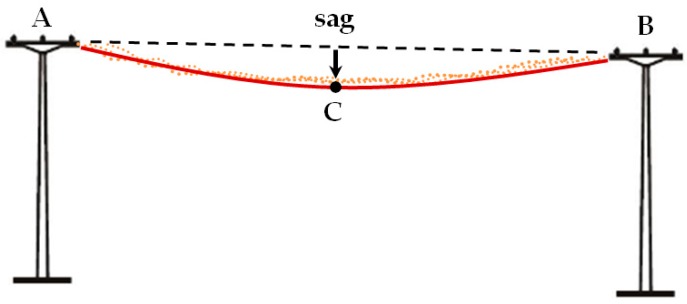
Schematic of line shape and parameters (sag).

The line length/span is measured as the distance between two points representing the anchoring of lines to the transmission towers and constitutes a reference line connecting points A and B (Figure 10). Power lines can be considered as free hanging lines, always deviating from the reference line. The deviation between the reference line connecting the line anchor points and the centre of the tension member cross-section, situated in the vertical direction, according to gravity, parallel to the Z coordinate axis, is referred to as sag. Sag is affected by temperature and has its maximum on hot days. The diameter is calculated as the average diameter of the cross-section of overhead cables measured on the denoised point clouds in different segments of the cables.

Every power line takes the form of a catenary, but the catenary is often replaced by a parabola in order to simplify the calculations. The obtained result is correct only if the tension members are fastened at similar heights and the spans are not too large. Then, based on the determined values of the coordinates of the A and B cable anchor points and the height of point C located in the middle of the span, the equation of the parabola can be determined. This is a simplified method, because we assume that the sag value reaches its maximum in the middle of the span between two pylons. Thus, for practical reasons, it is assumed that the cable sag is equal to the sag in the middle of the span reference line. The abovementioned parameters were determined on the basis of point clouds from UAV imagery and LiDAR data.

## 4. Experiment and Results

In this section, the experimental results for all data sets are reported. General information about the experiment flow chart can be found in Figure 6. Each “section” of the power lines between the pylons, regardless of the data set examined, was obtained during three or four flight strips as several photographs (80–96). Bundle adjustment was performed in Pix4Dmapper Pro software using dense image matching algorithms. As a result of the algorithm, point clouds were generated, each one characterised by considerable noise and discontinuity. Sample results are presented in Figure 1. Based on initial classification, the points were divided into ground, vegetation, buildings, pylons and lines. 

### 4.1. Point Cloud Filtering of Power Lines

**Figure 11:**
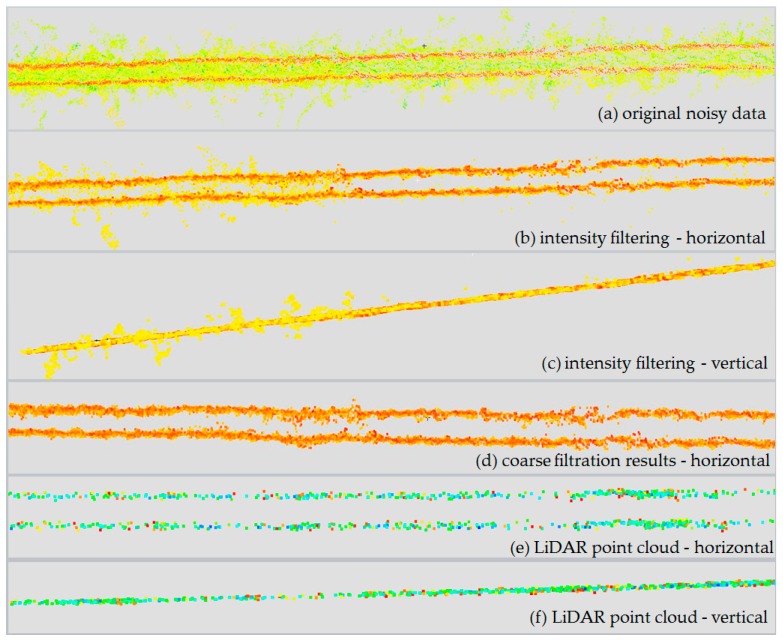
Results of different stages of coarse filtration: (**a**) original points, (**b**) intensity filtration—horizontal view, (**c**) intensity filtration—horizontal view, (**d**) result of filtering with intensity and geometric condition—horizontal view, (**e**), (**f**)—LiDAR point clouds—horizontal and vertical view.

In the process of coarse filtration, gross errors and outliers were eliminated. This was achieved with the use of the two-stage algorithm proposed in Section 3.3.1. This made it possible to set an appropriate intensity threshold and to eliminate the points that were not located in the immediate vicinity of the line points but which still had the required intensity. The process of coarse filtration is presented in Figure 11. 

In Figure 11a green and some yellow points represent the outliers—points subjected to segmentation under the intensity condition. The red and the part of orange coloured points represent real power lines points and some of the remaining noise. Figure 11b,c correspond to results of the first—intensity filtering. Visible points left over from the first stage were not eliminated until the geometric condition was applied. In Figure 11d final results of the whole coarse filtration (intensity and geometric) is shown. For comparison, Figure 11e,f present reference, corresponding LiDAR data with laser scanning intensity scale. It can be seen that LiDAR data are less noisy and have lower density. Statistical results of point clouds (minimum, maximum, mean and standard deviation) and threshold values *t* are presented below in Table 2. Shown in the ‘Filtration’ row is the percentage of points removed from the original point cloud after thresholding (intensity filtration).

As can be seen in Table 2, as a result of coarse filtration alone, the number of points representing gross errors in mixed pixels is lower by at least 65% in every case. In this way, most erroneous points that are difficult to eliminate from a point cloud generated from imagery were removed. The remaining points are difficult to classify as object/background points without further data analysis. 

### 4.2. Denoising with Wavelet Transform

**Figure 12:**
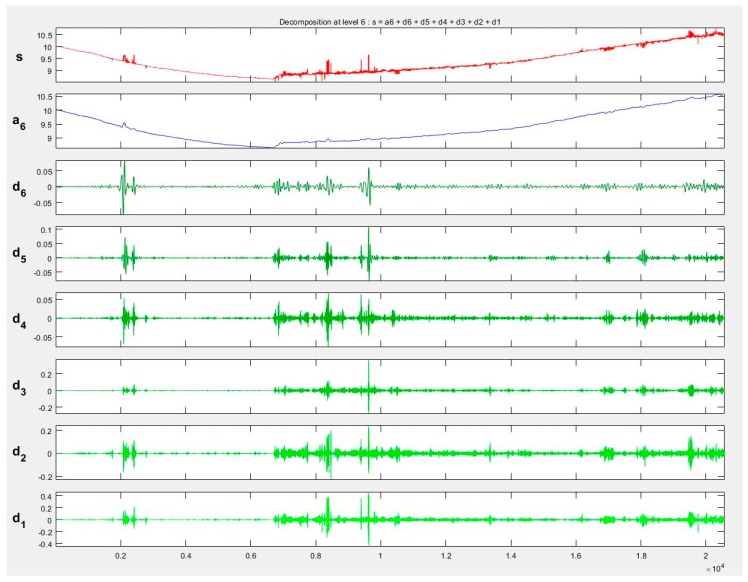
The fragment of decomposition of the Z coordinate with the DB6 L6 wavelet, s—original signal, a—approximation, d1–d6—decomposition details.

Pre-filtered point clouds representing power lines were divided into separate one-dimensional files (each containing a different coordinate). The points were oriented and segregated according to the Y coordinate to determine the horizontal axis of the coordinate system. This helped to separate signals representing only the Z coordinate, which was then subjected to wavelet analysis, because, as was already mentioned, this coordinate is the most important in the process of reconstructing power lines in 3D space and provides valuable information on factors such as, e.g., cable sag. The decomposition of signal for different families—Symlet, Haar, Daubeshies and Coiflet—was analysed. The selection of the family of the waves was related to their characteristics [54]. Finally, taking into account the conditions presented for example in [46], wavelet *db6* was chosen. Because of the length of the signals, the data was decomposed at level 6. Figure 12 shows the signal representing one of the decomposed lines *S3_L1_UAV* (data set III) of the Daubechies6 wavelet at level 6.

**Figure 13:**
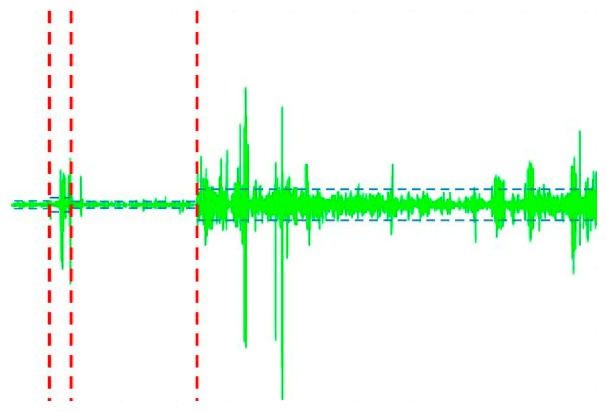
Sample thresholding in the denoising process for the d1 coefficient.

In all signal details, there were visible disturbances suggesting a certain nature of the data. In the case of power lines generated from UAV imagery, the changes in *d1* and *d2* details indicated point discontinuities or a lot of noise. The areas of these disturbances were reflected in the course of the signals or in the *a6* level approximation. In the next step, point clouds were denoised with the use of three algorithms: rigorous SURE, heuristic SURE and Fixed from Threshold. Value thresholding for these methods was conducted individually for every coefficient interval (Figure 13).

**Figure 14:**
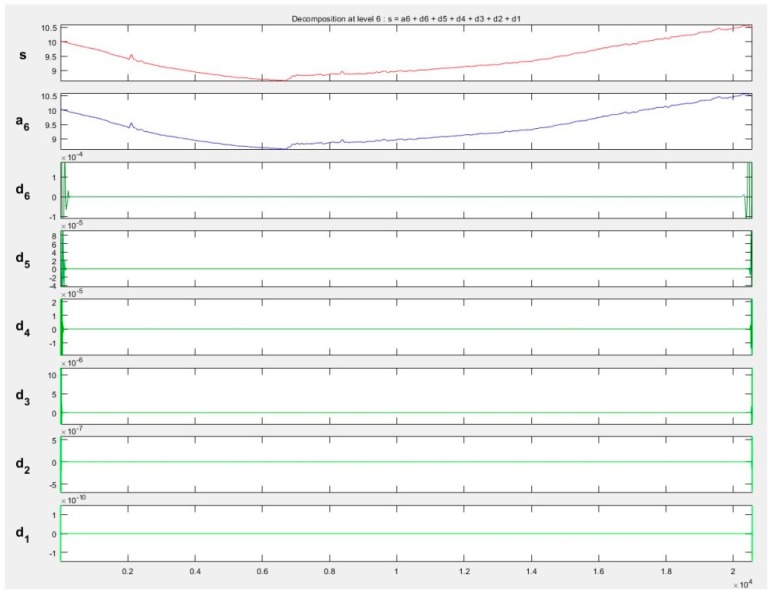
The fragment of decomposition of the Z denoised coordinate with the DB6 L6 wavelet, s—original signal, a—approximation, d1–d6—decomposition details.

The thresholds can be increased to keep only the highest values of the wavelet coefficients at each level. The interval-dependent strategy can be also defined separately and individually. Each level must be considered separately, and the thresholds adjusted. The current interval delimiters can also be propagated to all levels. Usually on the basis of signal statistics, intervals are proposed automatically. Since the variances for the three intervals are very different, the optimisation program easily detects the correct structure. Nevertheless, we can also define a dedicated number of intervals. The next step is to perform denoising. Decomposition of signal denoised with use of RSURE algorithm is presented in Figure 14.

**Figure 15:**
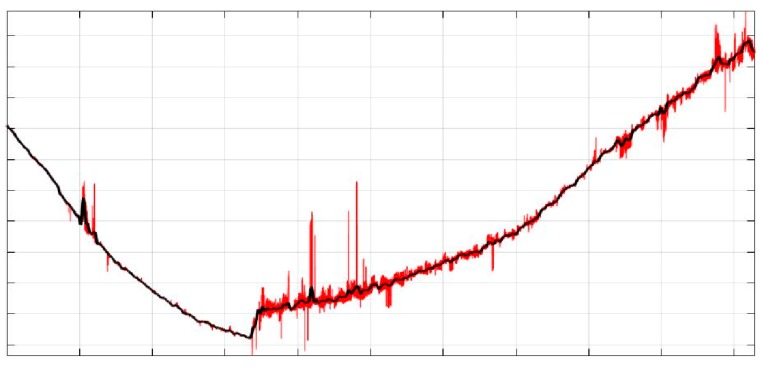
Z coordinate before and after denoising (red—original data, black—denoised data).

The process of denoising with the use of the RSURE algorithm had a radical impact on the data (bolded in Table 3). The coefficients that usually contain information on signal disturbances were very smooth (*d*1–*d*3). Then, the signal was reconstructed to its primary form. Theoretically, a denoised signal after reconstruction should be noiseless. The thresholding results are presented in Figure 15, where the black line represents the denoised signal and the red one shows the original signal. 

**Figure 16:**
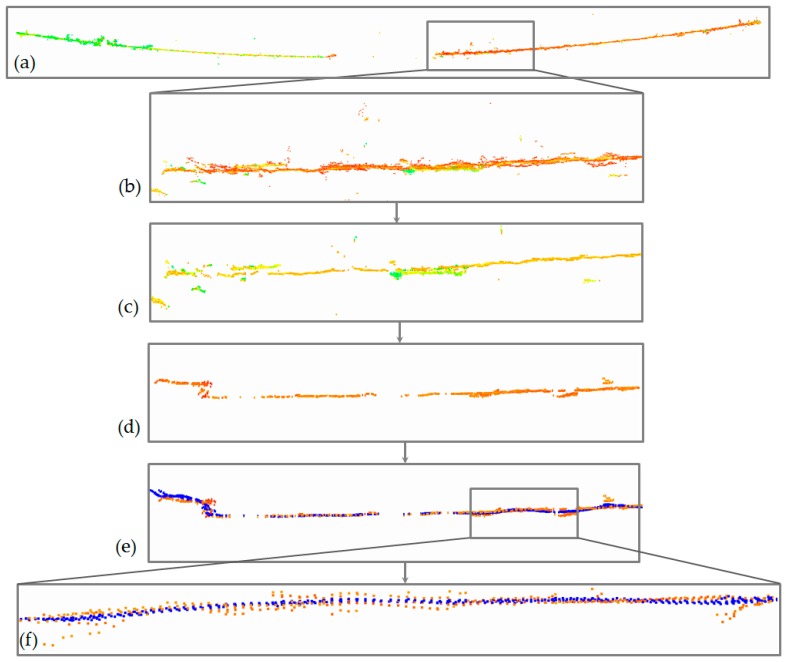
Power line (data set III) at different stages of the proposed algorithm: (**a**) the original point cloud, (**b**) close-up, (**c**) the results of coarse filtration with intensity condition, (**d**) the results of coarse filtration with geometric condition, (**e**) points denoised with the use of wavelet analysis (**f**) close-up of wavelet analysis results.

Similarly, decomposition and denoising were performed for all data sets using the proposed method. After reconstructing the denoised signal, the results obtained indicated an improved quality of point clouds. Presented in Figure 16 is a sample power line and its fragment filtered according to the proposed algorithm.

**Figure 17:**
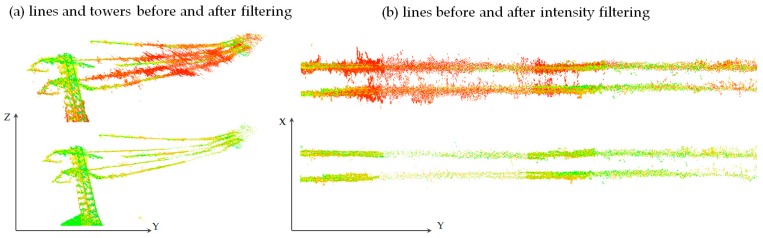
High-voltage power lines from UAV multi-images (**a**) lines and towers before and after intensity filtering, (**b**) lines before and after filtering.

Figure 16a shows the original point cloud with significant noise visible in the close-up in 16b. The following stages (Figure 16c,d) are the results of coarse filtration, first with the intensity condition, and then with the geometric condition. The final form of the data is presented in Figure 16e and the close-up in 16f, which also shows points denoised with the use of wavelet analysis (points in blue). Sample data before and after filtering is presented in Figure 17 for high-voltage power lines (data set I).

Figure 11, Figure 16 and Figure 17 represent point clouds that RGB colours were converted according to the scheme in Section 3.2. For LiDAR data—points are presented in original laser scanning intensity scale. Figure 17a,b show the elimination of points with a certain intensity (in this case marked with red). Apart from the visual analysis, statistical analysis of data correction is also important. The statistics for data before and after the filtration for coefficients (column *coeff.*) and data after the reconstruction (column *rec.*) are presented in Table 3. A more adequate and precise analysis is shown for the first level of details, named *Detail1*. This decomposition level was chosen because noise information is accumulated mostly in this detail. The parameters for residuals of the denoising *DB6* wavelet for *Detail1* are presented in the tables below, including both the original and denoised data. 

Significant changes are noticeable, especially in the mean absolute deviation (MAD), standard deviation (SD) and *L1norm* and *L2norm* values. When it comes to *Detail1*, the residual parameters *L1 norm*, *L2 norm* and *standard deviation* values are the highest for the original signal and decrease for consecutive threshold methods: *Fixed from Threshold* and both *SURE* algorithms. 

Similar results were obtained for the remaining test areas. Provided in Table 4 is a sample analysis of a high-voltage power line from data set II.

According to Equations (9) and (10), it can be noticed that the higher the values of *w1_dn_* and *w2_dn_* indexes, the more noiseless the data (bolded in Table 4). This also provides a good comparison of thresholding methods of denoising. In all cases, the RSURE method yields the most accurate data. The analysis was also conducted on the actual parameters of power lines and compared with the theoretical values and those obtained via laser scanning. Table 5 shows the values of point cloud resolution, line span and diameter before (ϕ_UAVo_) and after correction (ϕ_UAVc_), and sag before (**S_UAVc_**) and after correction (**S_UAVc_**). 

Table 5 shows the sag measurements for each power line computed by measuring the maximum difference in elevation from the reference line. The order of magnitude of the sag determined from UAV imagery point clouds was similar to the values obtained via laser scanning. There was also a significant improvement for data acquired with low-cost platforms. Data from UAV imagery point clouds were compared with the data from aerial (ALS) and terrestrial (TLS) laser scanning. Presented in Table 6 are the differences in sag values for UAV imagery (**S_UAVo_**—original, **S_UAVc_**—corrected) and laser scanning (LS) data. The largest differences are noticeable for resolution, cable diameter and point cloud resolution. There was less improvement for the line sag value. It is also interesting that sag values for the same line could be different in different seasons because of temperature. The sag value was usually smaller in winter than in summer. This is related to the properties of the power line material. These properties can be noticed for Line S2_L1_UAV in Table 5 and Table 7. 

As we are analysing different lines, each with different parameters, the comparison of accuracy is presented as a percentage in relation to the LiDAR data to determine the correction level. This comparison is presented in Table 6. 

With all the data obtained from a low-cost platform (set II and set III), there was a visible improvement in the accuracy of determining the line sag. These are calculations made against laser scanning data, which are assumed to carry a negligible error. The improvement in determining the sag value approached 10% when compared with the value obtained from laser scanning, while in the case of a professional platform (set I), the values did not improve or even slightly decreased (1–2%). However, there were significantly larger differences when comparing the other parameters, including cable diameter (Table 7).

In the case of the diameter, the actual (theoretical) value was known. In all sets, the values of the diameter after correction were the closest to the actual values. In one extreme case, the corrected diameter reached a value of 7 mm from the original 20 mm. Data filtration and denoising increased the accuracy by almost twofold. In the case of obtaining data for the same object in different seasons, and thus in different exposure conditions, there was also major improvement (50%).

The resulting accuracies are acceptable for power line inspection and monitoring, which typically requires an elevation accuracy of 10–20 cm. 

## 5. Discussion and Conclusions

This paper has presented an analysis of data obtained with both a professional and a low-cost unmanned platform. The aim of the study was to analyse the point clouds generated from UAV imagery with a potential application in the monitoring and inspection of power lines, especially with low-cost platforms. A precise power line model is a basic data requirement for further data processing: inspection or 3D modeling. Given the high level of noise in point clouds generated from UAV imagery, a method for improving their quality was proposed. From the experimental results, it is clear that improved point clouds result in an increased accuracy in power line parameter determination. Despite the “amount of noise”, the proposed method was shown to be effective. Thus, the advantage of the method developed is the independency from the SGM of SfM algorithms even with high noise. Sometimes, point clouds are not complete or more or less noisy. Therefore, the proposed coarse and precise filtering methodology was developed. The algorithm tolerates both noisy and very noisy point clouds in the input. Due to the other properties of proposed methods, very noisy point clouds might be used to represent a single power line more precisely (level of mixed pixel removal by coarse intensity filtering is about 65–80%). Furthermore, the proposed method can overcome the limitation of using low-cost UAV for low-voltage power lines mapping. 

Nevertheless, despite the improvement after the correction process, slight differences still existed between the UAV imagery point cloud and laser scanning. Two main limitations were observed in this study. Firstly, data sets used in research should be acquired using other low-cost platforms. However, the platform used in this study is one of the most common non-professional UAV platforms. The second limitation is the automatisation level of the last stage, i.e., the wavelet analysis. Nevertheless, the coarse filtration alone (the first part of the method) can be performed quickly and guarantees a high increase in data quality. Refinement with the use of the wavelet transform will only be necessary with less dense point clouds or when exposure conditions are unfavourable. 

In future work, the method automatisation level will be increased, and the data from other unmanned platforms will be studied. 

In future research work, the proposed method will be compared and integrated with existing power line reconstruction algorithms. Application of this method combined with other algorithms like RANSAC [9] or point cloud classification [5,7] would give very promising results. This would probably be even more effective than the separate use of them. 

## Figures and Tables

**Table 1:** Overview of the test data sets.

	Feature	Set I	Set II	Set III
Objects and area	Power line type Cable diameter (cm) Scene type	High-voltage (*ϕ_T_* = 30 mm) Open and forest area	Low-voltage (*ϕ_T_* = 6 mm) Rural area	Low-voltage (*ϕ_T_* = 28 mm) Urban area
Image and point clouds	Camera model GSD (cm) Flight height (m) Point cloud resolution (cm)	Sony Alpha 6000 4 cm 90 AGL 4	Sony RX100 0.3 23 m, 30 m 0.5–1.0	Sony RX100 0.5 28 m 0.6–1.2
**Set IV** Reference data type	Laser scanning model and type	Riegl VUX-1UAV (UAV-ALS)	Leica P40 (TLS)	Leica P40 (TLS)

**Table 2:** Intensity and filtration statistics for chosen lines from all data sets.

	SET I (Summer)	SET II (Summer)	SET II (Winter)	SET III (Autumn)
Minimum	0.499	0.499	0.499	0.499
Maximum	0.560	0.560	0.560	0.560
Mean	0.509	0.523	0.529	0.512
SD	0.019	0.013	0.015	0.009
Max. SD	0.031	0.031	0.031	0.031
*K* coefficient	0.20	0.15	0.20	0.07
Threshold value *t*	0.522	0.523	0.525	0.513
Filtration	68%	67%	75%	84%

**Table 3:** Statistics for the coefficients and reconstruction of the original and denoised signals for a power line (data set III).

	Original Data		Fixed from Threshold		RSURE		HSURE	
	*Coeff.*	*Rec.*	*Coeff.*	*Rec.*	*Coeff.*	*Rec.*	*Coeff.*	*Rec.*
*SD*	0.024	0.020	0.018	0.012	4.769 × 10^−07^	1.972 × 10^−07^	0.018	0.013
*MAD*	0.005	0.009	0.002	0.002	1.045 × 10^−08^	6.24 × 10^−09^	0.002	0.002
*L1 norm*	129.4	182.6	19.55	33.93	0.0001	0.0001	22.09	38.16
*L2 norm*	2.879	2.879	1.808	1.808	4.837 × 10^−05^	0.0001	1.877	1.877
*Max norm*	0.624	0.459	0.567	0.389	3.645 × 10^−05^	0.0001	0.574	0.398
*w1_dn_*			6.0	4.6	**82.7**	**105.2**	5.4	4.2
*w2_dn_*			8.8	7.5	**50,201**	**101,935**	8.1	7.0

**Table 4:** Statistics for the coefficients and reconstruction of the original and denoised signal for a power line (data set II).

	Original Data		Fixed from Threshold		RSURE		HSURE	
	*Coeff.*	*Rec.*	*Coeff.*	*Rec.*	*Coeff.*	*Rec.*	*Coeff.*	*Rec*.
*SD*	0.004	0.003	0.002	0.001566	0.001991	0.0014	0.0022	0.0015
*MAD*	0.002	0.002	0.0003	0.000225	0.0002494	0.0002	0.0003	0.0002
*L1 norm*	25.3	35.3	2.77	4.69	2.44	4.21	2.77	4.69
*L2 norm*	0.447	0.447	0.226	0.226	0.203	0.203	0.226	0.226
*Max norm*	0.086	0.077	0.078	0.066	0.077	0.066	0.077	0.066
*w1_dn_*			5.8	5.6	**6.6**	**6.3**	5.8	5.6
*w2_dn_*			18.2	20.3	**20.4**	**22.6**	18.2	20.3

**Table 5:** Parameters of selected lines before and after correction.

	Line	Point Cloud Resolution (mm)	Span (m)	Theor. Cable Diameter ϕ_T_ (mm)	Cable Diameter ϕ_UAVo_ (mm)	Cable Diameter Corrected ϕ_UAVc_ (mm)	Sag S_UAVo_ (m)	Sag Corrected S_UAVc_ (m)
*SET I*	S1_L1_ALS	38	292.69	30	46	-	5.52	-
	S1_L1_UAV	43	291.88		96	64	5.71	5.61
	S1_L2_ALS	38	291.19		65	-	8.10	-
	S1_L2_UAV	43	291.25		88	58	8.24	8.31
*SET II*	S2_L3_TLS	6	48.94	6	10	-	0.26	-
	S2_L3_UAV	4	48.41		10	8	0.22	0.25
	S2_L1_TLS (winter)	4	44.52		13	-	0.47	-
	S2_L1_UAV (summer)	10	44.86		26	15	0.76	0.69
	S2_L1_UAV (winter)	5	43.78		12	10	0.51	0.46
*SET III*	S3_L1_TLS	7	40.62	28	30	-	1.61	-
	S3_L1_UAV	11	40.62		42	29	1.58	1.63

**Table 6:** Comparison of line sag before and after correction for UAV imagery and laser scanning.

	SET I		SET II			SET III
Sag Delta [m]	S1_L1	S1_L2	S2_L3	S2_L1 (Winter)	S2_L1 (Summer)	S3_L1
**S_UAVo_**–**S_LS_**	0.19	0.14	0.03	0.04		0.03
**S_UAVc–_S_LS_**	0.09	0.20	0.01	0.01		0.02
**S_UAVo_**–**S_UAVc_**	0.09	0.07	0.02	0.04	0.07	0.05
Correction level	2%	1%	10%	10%	9%	3%

**Table 7:** Comparison of diameter before and after correction for UAV imagery and laser scanning data.

Diameter Delta (mm)	S1_L1	S1_L2	S2_L3	S2_L1 (Winter)	S2_L1 (Summer)	S3_L1
ϕ_UAVo_–ϕ_T_	66	58	4	6	20	14
ϕ_UAVc_–ϕ_T_	34	28	2	4	9	1
ϕ_LS_–ϕ_T_	16	35	4	7	7	2
